# TaxaGloss - A Glossary and Translation Tool for Biodiversity Studies

**DOI:** 10.3897/BDJ.4.e10732

**Published:** 2016-12-21

**Authors:** Rachel Collin, Suzanne Fredericq, D. Wilson Freshwater, Edward Gilbert, Maycol Madrid, Svetlana Maslakova, Maria Pia Miglietta, Rosana M. Rocha, Estefanía Rodríguez, Robert W. Thacker

**Affiliations:** 2 Department of Biology, University of Louisiana at Lafayette, Lafayette, United States of America; 3 Center for Marine Science, University of North Carolina at Wilmington, Wilmington, United States of America; 4 School of Life Science, Arizona State University, Tempe, United States of America; 1 Smithsonian Tropical Research Institute, Panama City, Panama; 5 Oregon Institute of Marine Biology, University of Oregon, Charleston, United States of America; 6 Department of Marine Biology, Texas A&M University at Galveston, Galveston, United States of America; 7 Department of Zoology, Universidade Federal do Paraná, Curitiba, Brazil; 8 Division of Invertebrate Zoology, American Museum of Natural History, New York, United States of America; 9 Department of Ecology and Evolution, Stony Brook University, Stony Brook, United States of America

**Keywords:** species identification, taxonomy, systematics, marine invertebrate, biodiversity, phenotype, phenoscape, ontology, Symbiota, seaweed

## Abstract

**Background:**

Correctly identifying organisms is key to most biological research, and is especially critical in areas of biodiversity and conservation. Yet it remains one of the greatest challenges when studying all but the few well-established model systems. The challenge is in part due to the fact that most species have yet to be described, vanishing taxonomic expertise and the relative inaccessibility of taxonomic information. Furthermore, identification keys and other taxonomic resources are based on complex, taxon-specific vocabularies used to describe important morphological characters. Using these resources is made difficult by the fact that taxonomic documentation of the world's biodiversity is an international endeavour, and keys and field guides are not always available in the practitioner's native language.

**New information:**

To address this challenge, we have developed a publicly available on-line illustrated multilingual glossary and translation tool for technical taxonomic terms using the Symbiota Software Project biodiversity platform. Illustrations, photographs and translations have been sourced from the global community of taxonomists working with marine invertebrates and seaweeds. These can be used as single-language illustrated glossaries or to make customized translation tables. The glossary has been launched with terms and illustrations of seaweeds, tunicates, sponges, hydrozoans, sea anemones, and nemerteans, and already includes translations into seven languages for some groups. Additional translations and development of terms for more taxa are underway, but the ultimate utility of this tool depends on active participation of the international taxonomic community.

## Introduction

The correct identification of organisms is vital to all fields in biology. Species identification is a particular challenge for those working in biodiversity science where researchers and conservation practitioners often encounter a broad diversity of species from a variety of taxa. These often include rare, poorly known and undescribed organisms as well as those that are outside the researcher's area of expertise. In these cases workers must draw on a scattered literature of taxonomic keys, field guides, and primary taxonomic literature to assist with identifications. This literature includes complex, taxon-specific vocabularies, and is inaccessible to those who are not familiar with the technical terms. For many groups glossaries explaining these terms are difficult to find and are commonly published in journals with limited circulation. Few glossaries are available in a searchable online format and they are seldom illustrated. In addition, researchers must also often consult species descriptions or taxonomic monographs in multiple languages, but glossaries are available in a few languages, mainly English, French and German.

**Figure 1.**
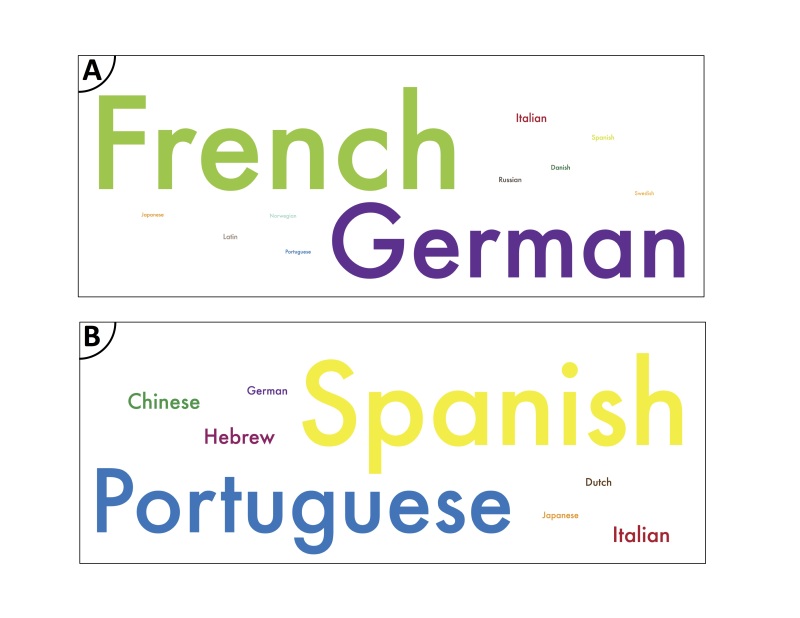
A word cloud showing the numbers of (a) languages represented in the literature on tunicates from the Ascidiacea World Database and (b) participants in four 2-week courses on tunicate biology and systematics at the Bocas del Toro Research Station between 2006 and 2014. English was the dominant language and is excluded from figure for clarity. **Publications**: English - 408; German - 134; French - 181; Italian - 16; Russian - 10; Spanish - 4; Portuguese - 1; Latin -10; Norwegian - 1; Danish - 10; Japanese - 1; Swedish - 1. **Participants**: English -28; Spanish -16; Portuguese - 10; Dutch -1; Chinese - 2; German -1; Italian -2; Hebrew - 2; Japanese - 1.

Biologists working with ascidians provide an example of the scope of this linguistic challenge. The 780 publications available in the source list of the Ascidiacea World Database as of October 2015 (Shenkar et al. 2015) are written in 12 languages. While slightly more than half have been published in English, a significant number are in German or French (Fig. 1a). The primary tunicate taxonomists working and training students today are based in Brazil, Israel, Spain, Japan and the United States. The community of students and taxonomists in training is even more linguistically diverse. Four courses in tunicate taxonomy held between 2006 and 2014 at the Smithsonian Tropical Research Institute's Bocas del Toro Research Station hosted students from 21 countries including native speakers of eight languages (Fig. 1b). Expert instructors participating in the same four courses came from five countries with four native languages. Similar examples could be drawn from a number of other taxa.

The diversity of languages in use in the taxonomic literature as well as among the current practitioners and students of a taxon means that translations between a large number of language pairs are needed. To a large extent GoogleTranslate can be used to obtain translations of background text in scientific publications between most pairs of languages, and taxon names are given in multiple languages in the World Register of Marine Species (Costello et al. 2013). However, species descriptions and keys, which are largely based on morphology and involve a number of highly specialized taxon-specific terms generally fail to translate adequately with such online tools. The idiosyncratic nature of the taxonomic literature means that in some cases researchers may have to translate technical terms by going through a third language, if they can find any translated glossaries. To aid in this process we developed TaxaGloss — a multilingual illustrated glossary and translation tool. We hope that it will increase access to definitions of morphological terms and aid taxonomists, biodiversity researchers, students and others in their understanding of the taxonomic literature.

## Design and Implementation

**Figure 2.**
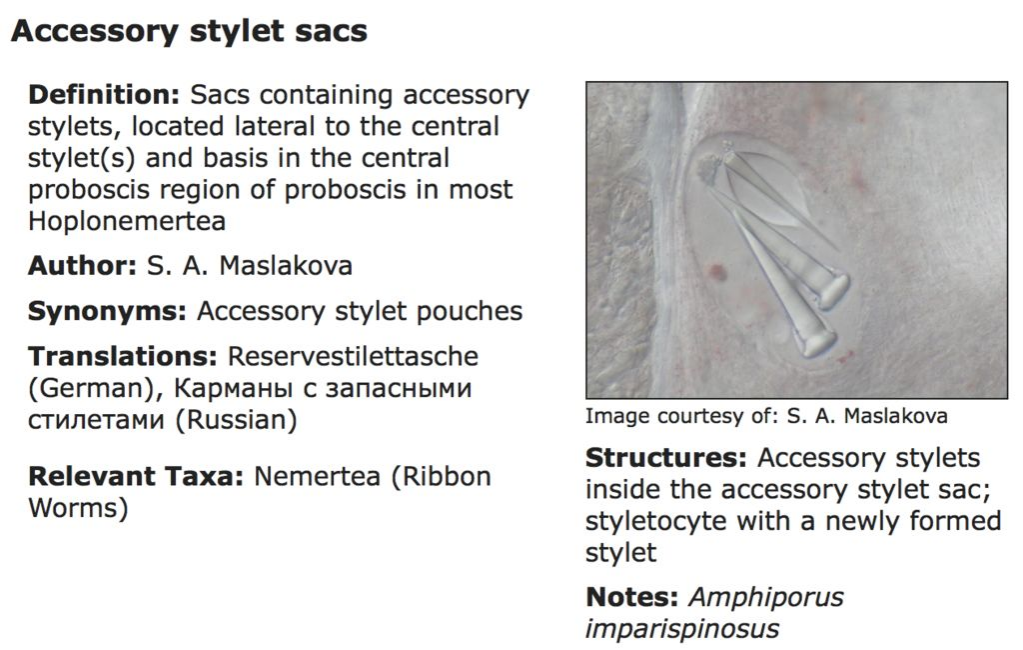
The TaxaGloss display of a single term from the Nemertea.

**Figure 3.**
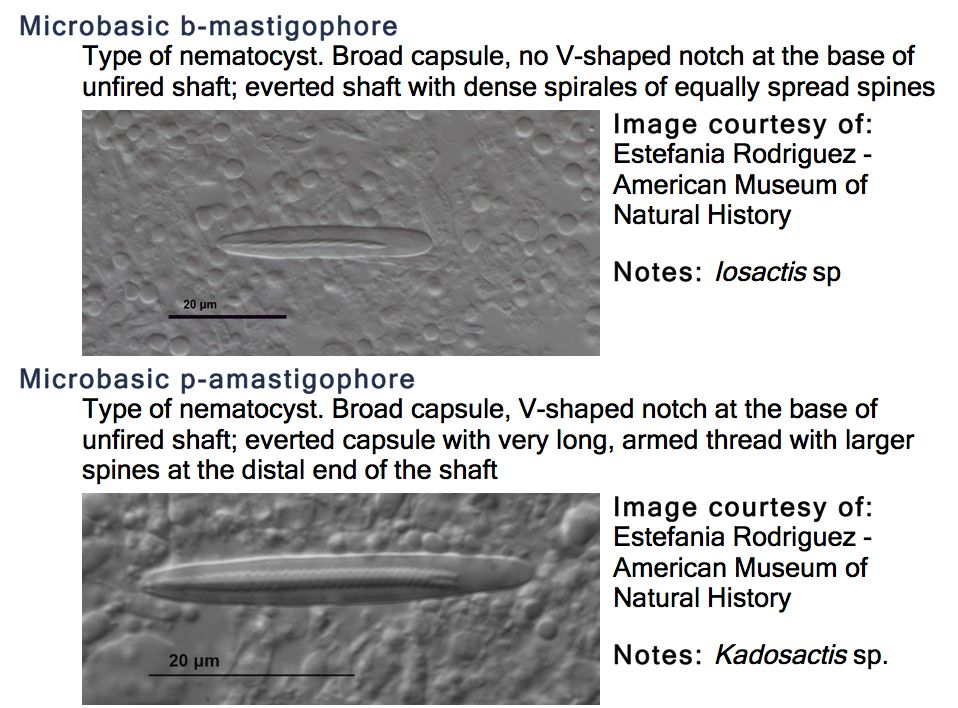
The TaxaGloss display of the single-language glossary output, showing the term, definition, and illustration for two Actiniaria terms.

**Figure 4.**
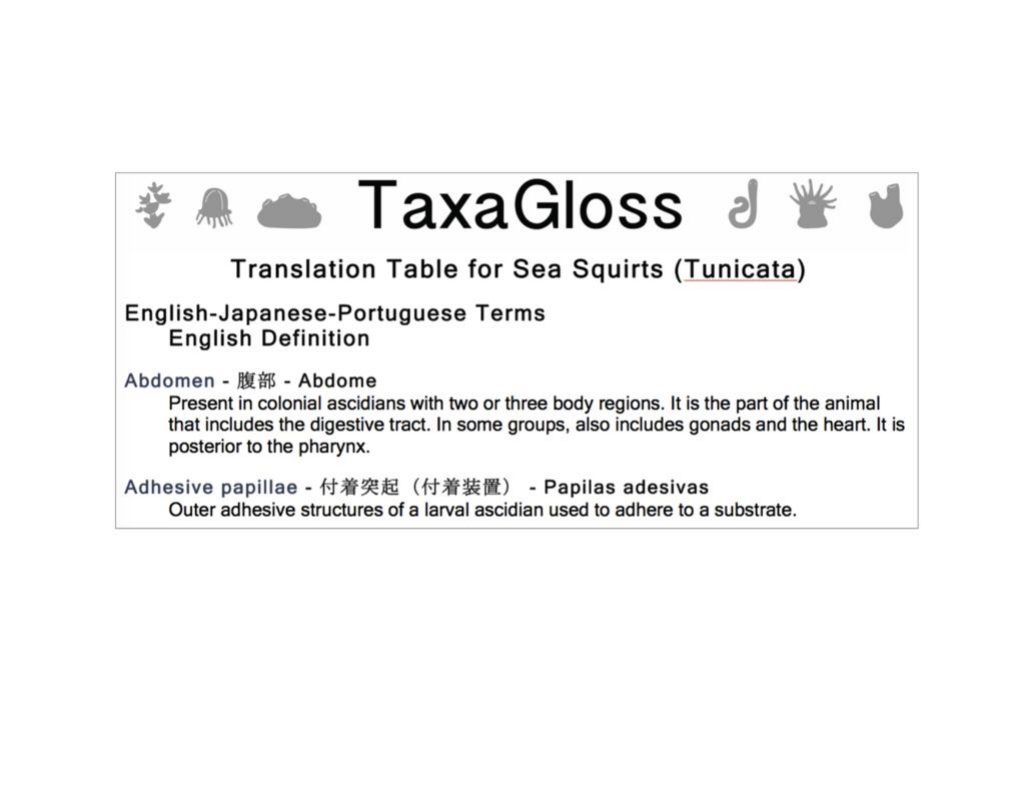
The TaxaGloss display of the custom translation table output showing a translation for two Tunicata terms between English, Japanese and Portuguese with a definition in English.

TaxaGloss has been designed as a PHP/JavaScript web application integrated into the Symbiota software platform (Gries et al. 2014). Symbiota follows the Open Source paradigm (Gries et al. 2014) allowing the application to be easily implemented within any Symbiota portal. TaxaGloss is currently implemented as part of the Marine Life of Panama Portal. Data consisting of terms and definitions in any language, images, citations and links to formal ontologies can be entered manually or batch processed. Data can be used to produce output for a single term, a single-language illustrated glossary, or a custom translation table (Figs 2, 3, 4). Single language glossaries contain the definition, and an illustration (optional) for all terms pertaining to a selected taxon as well as citations and acknowledgements (Fig. 3). Custom translation tables, with up to four data fields, can be created by selecting terms and/or definitions in multiple languages (Fig. 4).

TaxaGloss currently includes glossaries for six marine taxa: macroalgae (divisions Rhodophyta and Chlorophyta, and class Phaeophyceae), sponges (phylum Porifera), hydroids (phylum Cnidaria, class Hydrozoa), sea anemones (phylum Cnidaria, class Anthozoa, order Actinaria), nemerteans (phylum Nemertea), and tunicates (phylum Tunicata, class Ascidiacea). These foundation taxa were selected because they are the focal groups in the BocasARTS project and they have been or will be subjects of taxonomy training courses as part of the Smithsonian Tropical Research Institute’s (STRI) Training in Tropical Taxonomy program. Each glossary began with a core list of terms and definitions complied by the taxon editor for each group. These lists include original content as well as terms or definitions drawn from previously published lists of terms (Table 1) and online glossaries. Translations of the terms and the definitions were provided by multilingual taxon editors or solicited from qualified colleagues and collaborators. Images were provided by taxon editors, colleagues and collaborators, and students participating in the taxonomy training program. Finally, a scientific illustrator was commissioned to produce bauplan illustrations and schematics for a subset of the terms. At its launch in 2016, TaxaGloss included over 1,300 English terms, 500 of which are connected to a photograph or illustration (Table 1). Depending on the taxon, each term had been translated into 2-7 languages (Table 1).

## Utility, Discussion and Future Prospects

TaxaGloss offers an open-access, web-based, illustrated glossary for technical terms used in biodiversity studies and systematics. This information is otherwise scattered throughout an often difficult to access literature. By increasing accessibility to this information TaxaGloss will provide an entrée to the literature for researchers and students who do not have access to mentors with expertise in their taxon of interest. Easy access to a single glossary may also help to standardize usage of obscure terms, as the inconsistent use of terms that plague the taxonomic literature in some groups may stem from lack of accessible reference sources.

Although English is the current language of taxonomy and systematics, this has not always been the case. Much useful data and many original species descriptions have been published in other European languages and for some taxa there is a significant body of literature in Russian and a number of Asian languages. Today, many of the users of taxonomic and biodiversity data are not native English speakers. This provides a challenge for working taxonomists as well as for training local taxonomists and parataxonomists who may have expert knowledge of the organisms but may face a linguistic impediment to understanding the literature. We expect that TaxaGloss will assist such practitioners in their daily work and in training students. Primarily it provides an open-access, user-friendly illustrated list of terms, accessible in a number of languages. For many taxa there are glossaries published in few languages, which are available only in obscure publications that may not be easily accessible. For other taxa no such glossaries are currently available. The illustrations may be particularly valuable for helping undergraduate students understand the internal anatomy of marine invertebrates in lab classes that involve dissections. Students in R. M. Rocha's laboratory in Brazil have already found the glossary to be useful in their daily work. They access it on their smartphones and tablet computers as they work their way through dissections. Secondarily, the translation table tool will facilitate translations of the literature, and will possibly result in a more congruent and homogeneous use of taxonomic terms. We also anticipate this electronic tool will facilitate scientists at any stage of their career, and especially younger generations of researchers in countries with few or no remaining taxonomic experts, in understudied groups such as those available on TaxaGloss.

## Figures and Tables

**Table 1.** Summary of taxa and sources in the first version of TaxaGloss.

**Group** **(Taxon Editors)**	**Number of Terms**	**Number of Figures**	**Languages**	**Primary References for Terms**
**Seaweeds** **(Fredericq and Freshwater)**	254	67	English, Spanish, Portuguese (partial), Thai, French, Dutch	Dawes and Mathieson 2008, Hine 1976, Schneider and Searles 1991, Stearn 1992
**Sponges** **(Thacker)**	540	406	English, Portuguese (partial), Spanish (partial)	Boury-Esnault and Ruetzler 2007, Thacker et al. 2014, Hajdu et al. 2011
**Hydroids** **(Miglietta)**	365	0	English, Spanish, Italian, Portuguese	Bouillon et al. 2006
**Sea Anemones** **(Rodríguez)**	67	26	English, Spanish	Häussermann and Försterra 2009, Carlgren 1949
**Nemerteans** **(Maslakova)**	31	58	English, German, Russian	Chernyshev 2011, Bürger 1895, Roe et al. 2007
**Tunicates** **(Rocha)**	130	185	English, Spanish, Portuguese, French, German, Hebrew, Japanese	Kott 1985, Kott 1990, Kott 1992, Kott 2001, Nishikawa 1995, Ishikawa et al. 1986, Berrill 1950

## References

[B3382321] Berrill N. J. (1950). The Tunicata. With an account of the British species..

[B3336522] Bouillon J, Gravili C, Pagès F, Gili J-M, Boero F (2006). An introduction to Hydrozoa. Mémoires du Muséum National d'Histoire Naturelle 1936.

[B3336512] Boury-Esnault N, Ruetzler K (2007). Thesaurus of Sponge Morphology.. Smithsonian Contributions to Zoology.

[B3336533] Bürger O (1895). Die Nemertinen des Golfes von Neapel und der angrenzenden Meeres-Abschnitte. Fauna Flora Golf Neapel.

[B3382352] Carlgren O. (1949). A survey of Ptychodactiaria, Corallimorpharia and Actiniaria. Kungliga Svenska Vetenskapsakademiens Handlingar.

[B3336552] Chernyshev AV (2011). Comparative morphology, systematics and phylogeny of nemerteans.

[B3493095] Costello Mark J., Bouchet Philippe, Boxshall Geoff, Fauchald Kristian, Gordon Dennis, Hoeksema Bert W., Poore Gary C. B., van Soest Rob W. M., Stöhr Sabine, Walter T. Chad, Vanhoorne Bart, Decock Wim, Appeltans Ward (2013). Global Coordination and Standardisation in Marine Biodiversity through the World Register of Marine Species (WoRMS) and Related Databases. PLoS ONE.

[B3336561] Dawes CJ, Mathieson AC (2008). The Seaweeds of Florida.

[B3336342] Gries Corinna, Gilbert Edward E, Franz Nico M (2014). Symbiota - A virtual platform for creating voucher-based biodiversity information communities.. Biodiversity data journal.

[B3337885] Hajdu E, Peixinho S, Fernandez JCC (2011). Esponjas Marinhas da Bahia: Guia de campo e laboratório.

[B3337894] Häussermann V, Försterra G (2009). Marine Benthic Fauna of Chilean Patagonia.

[B3337903] Hine AE (1976). A Glossary of Phycological Terms for Students of Marine Macroalgae - An Aid for Interpreting Keys and Descriptions Relating to the Four Divisions of Macroalgae: Cyanopyta, Chlorophyta, Phaeophyta, and Rhodophyta.

[B3382330] Ishikawa M., Kawamura K., Taneda Y., Nakauchi M., Nishikawa T., Mukai H., Watanabe H. (1986). Systematic Zoology Vol. 8 Part 2 Hemichordata and Prochordata..

[B3337966] Kott P (1985). The Australian Ascidiacea part 1, Phlebobranchia and Stolidobranchia. Memoirs of the Queensland Museum.

[B3337977] Kott P (1990). The Australian Ascidiacea, part 2, Aplousobranchia (1). Memoirs of the Queensland Museum.

[B3337987] Kott P (1992). The Australian Ascidiacea part 3, Aplousobranchia (2). Memoirs of the Queensland Museum.

[B3338006] Kott P (2001). The australian Ascidiacea Part 4, Aplousobranchia (3), Didemnidae.. Memoirs of the Queensland Museum.

[B3382307] Nishikawa T., Nishimura S. (1995). Chordata. Guide to Seashore Animals of Japan with Color Pictures and Keys Vol. II.

[B3337921] Roe P, Norenburg JL, Maslakova SA, Carlton JT (2007). Nemertea. The Light and Smith Manual: Intertidal Invertebrates from Central California to Oregon 4th Edition.

[B3337935] Schneider CW, Searles RB (1991). Seaweeds of the Southeastern United States - Cape Hatteras to Cape Canaveral.

[B3337944] Shenkar Noa, Gittenberger Arjan, Lambert Gretchen, Rius Marc, Rocha Rosana Moreira, Swalla Billie J, Turon Xavier Ascidiacea World Database.. http://www.marinespecies.org/ascidiacea.

[B3337956] Stearn WT (1992). Botanical Latin.

[B3336319] Thacker Robert W, Díaz Maria, Kerner Adeline, Vignes-Lebbe Régine, Segerdell Erik, Haendel Melissa A, Mungall Christopher J (2014). The Porifera Ontology (PORO): enhancing sponge systematics with an anatomy ontology. Journal of Biomedical Semantics.

